# Quantitative oncogene-mapping within malignant tumors through Multi-parameter MRI based on RNA-triggered nanoprobes

**DOI:** 10.1016/j.mtbio.2026.102936

**Published:** 2026-02-13

**Authors:** Wenyue Li, Runjie Wang, Xinyi Zhang, Shuai Wu, Peisen Zhang, Hongxiang Feng, Yue Lan, Zhuo Ao, Yi Hou

**Affiliations:** a Department of Rehabilitation Medicine, School of Medicine, the Second Affiliated Hospital of South China University of Technology (Guangzhou First People's Hospital), Guangzhou, 510180, China; b College of Materials Science and Engineering, and College of Life Science and Technology, Beijing University of Chemical Technology, Beijing, 100029, China; c CAS Center for Excellence in Nanoscience, National Center for Nanoscience and Technology, Beijing, 100190, China; d Department of Thoracic Surgery, China-Japan Friendship Hospital, Beijing, 100029, China

**Keywords:** Magnetic resonance imaging, Magnetic resonance tuning, Multi-parameter, Tumor-associated miRNA, Oncogene-mapping

## Abstract

Genomic instability is the important foundation and feature of cancer; hence, the precise oncogene detection is crucial for early diagnosis and pathological analysis of tumors. However, the *in vivo* oncogene-imaging is confronted with great challenge, due to the extremely low copies of oncogene in cancer cells. Herein, we proposed a strategy of *T*_1_/*T*_2_ magnetic resonance imaging (MRI) based on magnetic resonance tuning (MRET) to analyze tumor-associated miRNA quantitatively. The superparamagnetic quencher Fe_3_O_4_ nanoparticles and the paramagnetic enhancer Gd-DTPA were integrated by the DNA linker. The AS1411 aptamer was embedded at the end of the DNA linker to effectively target nucleolins that are overexpressed in tumor cells. As hybridization with targeted miRNA, the Gd^3+^ labeled tumor-associated nucleotide sequence is released, enhancing *T*_1_ signals and enabling specific localization and quantification of the targeted miRNA. The MRET effect was verified *in vitro* through mixing with varying concentrations of miR-21. Enhanced *T*_2_ signals and activated *T*_1_ signals were visualized in 4T1 or CT26 subcutaneous tumor models *in vivo*. Ultimately, the quantitative relation between MRI signals and local miR-21 concentration was established *in vivo*. These results indicated the potential of nanoprobes for tumor-related genes diagnosis and quantification, further affirming the possibility of prompt and precise tumor diagnosis.

## Highlights

• RNA-triggered nanoprobes were developed via MRET strategy upon RNA hybridization.
• The nanoprobes enabled *in vivo* tumor detection through *T*_1_/*T*_2_ dual-modality MRI.
• The nanoprobes enabled *in vivo* quantitative visualization of miR-21.

## Graphical abstract

The *T*_1_ signals of magnetic resonance imaging (MRI) nanoprobes were activated via magnetic resonance tuning (MRET), enabling quantitative visualization the cancer-related miR-21 *in vivo*.

**Figure:**
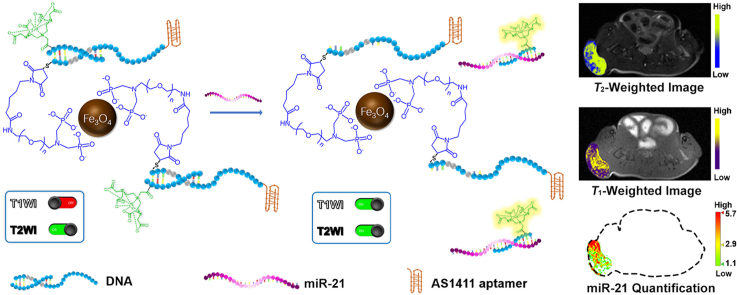

## 1 Introduction

Malignant tumor has become one of the leading diseases threatening the public health worldwide [[1], [2], [3]]. However, there is still a lack of effective and accurate clinical methods for early diagnosis, due to the complex of the tumor procession [4]. Genomic instability is a fundamental and critical characteristic of cancer initiation and progression [[5], [6], [7]]. The malignant biological behaviors of tumors, including occurrence, development, and metastasis, involve a complex pathophysiological process induced by multiple factors, with the participation of various genes at different stages. This process includes the activation of oncogenes and the inactivation of tumor suppressor genes, which ultimately lead to dysregulation of cell proliferation, differentiation, and apoptosis. However, there are significant obstacles in identifying oncogenes *in vivo* in malignant cells and tissues. For example, low abundance and dynamic changes of oncogenes expression levels hinder the development of genetic diagnostics [[8], [9], [10]]. Furthermore, the oncogene yields with direct imaging show low signal intensity, making it difficult to reach the detection limits for imaging diagnosis.

Tumor-associated RNA is involved in the expression and regulation of oncogenes, and the copy number of tumor-related RNAs is significantly higher than that of oncogenes.[11]. Therefore, tumor-related RNAs can serve as the key molecular targets associated with malignancy, promisingly achieving the specific imaging of revealing tumor characteristics at the molecular level. MicroRNAs (miRNAs) are a class of short single-stranded endogenous non-coding RNA molecules with a length of 21-23 nucleotides [12]. Most of researches have suggested that the abnormal expression of miRNA is closely related to many types of cancers. For instance, MicroRNA-21(miR-21) is upregulated in the breast carcinoma, which can be as a crucial player in tumor development and progression [13,14]. On this basis, miRNA can serve as potential diagnostic biomarkers with clinical practice prospect for early cancer theranostics. The quantitative expression of miRNA is greatly significant for understanding miRNA-related physiological and pathological processes, and developing miRNA-based therapeutic approaches [15]. Up to now, there exist several studies [16,17] about miRNA-targeting nanoprobes based on different DNA structures [[18], [19], [20]] and nanomaterials [21,22] for tumor imaging, mostly for *in vitro* detection without exact quantification. It is quite challenging to deal with quantitative miRNA visualization *in vivo*.

The magnetic resonance tuning (MRET) effect enables quantitative miRNA analysis *in vivo*. In 2017, Jinwoo Cheon et al. defined nanoscale distance-dependent phenomena of *T*_2_ and *T*_1_ contrast agents as MRET [23,24]. MRET involves two components: a superparamagnetic ‘quencher’ (Q) and a paramagnetic ‘enhancer’ (E), with their nanoscale separation (d) governing MRET efficacy. When E and Q are separated (d_1_> d_c_, critical distance), E's fast electron spin fluctuation accelerates water proton relaxation, yielding a strong *T*_1_ MRI signal. Conversely, when E approaches Q (d_2_< d_c_), spin fluctuation slows, failing to promote relaxation and producing a weak *T*_1_ signal. The nanoscale distance-dependent phenomena can effectively eliminate high background signal interference. Thus, quantitative visualization of miRNAs *in vivo* can be achieved via the MRET effect with unnecessary interference.

**Scheme 1:**
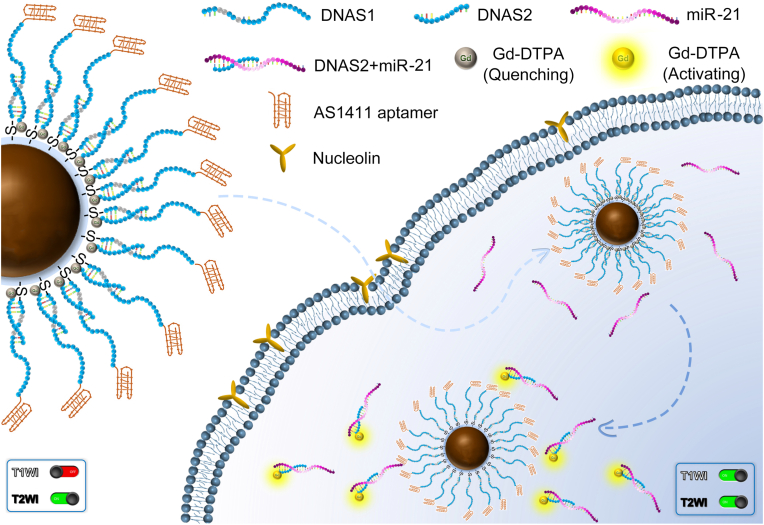
Schematic illustration of positional and quantitative strategy *in vivo* of nanoprobes.

The current work aims to develop an *in vivo* quantitative oncogene-mapping strategy with responsive *T*_1_/*T*_2_ magnetic resonance imaging (MRI) nanoprobes. Relying on the MRET effect between the superparamagnetic Fe_3_O_4_ nanoparticles and the paramagnetic Gd-DTPA, the nanoprobes can enable the weak-to-strong *T*_1_ signal transition. This capability allows the nanoprobes to avoid background interference and specifically identify and localize miR-21, which is upregulated in breast carcinoma. The constructed diagram of nanoprobes is shown in Fig. S1. The nanoprobes link Fe_3_O_4_ nanoparticles to Gd-DTPA through the bridging effect of hybridized nucleic acid strands, and are modified with the AS1411 aptamers that can specifically target nucleolins highly overexpressed in tumor cells. The *in vivo* functional mechanism of nanoprobes is illustrated in Scheme 1. After administration, the AS1411 aptamer carries nanoprobes to target the nucleolin, and with nucleolin-mediated transmembrane transport, the nanoprobes can enter the tumor cells, where hybridization reactions between the miR-21 and the complementary DNA sequence take place. The *T*_1_ signals of paramagnetic Gd-DTPA, previously restricted by superparamagnetic Fe_3_O_4_ nanoparticles, are finally activated upon the target miR-21 recognition. The *T*_1_ MRI signals triggered in this process can guide identification and quantitation of miR-21. Specifically, by leveraging the *in vitro* relationship between the change in longitudinal relaxivity (Δ*R*_1_) and miR-21 concentration, effective quantitative analysis of miR-21 *in vivo* can be achieved. This strategy allows for the precise localization and analysis of early-stage cancerous microlesions, and provides a warning for metastasis and prognosis evaluation, thus having significant scientific value and positive social implications. It is expected to greatly enhance the detection sensitivity and accuracy of early-stage cancer microlesions and provide a basis for tumor treatment and prognosis.

## 2 Results

### 2.1 Construction and characterization of nanoprobes

**Fig. 1:**
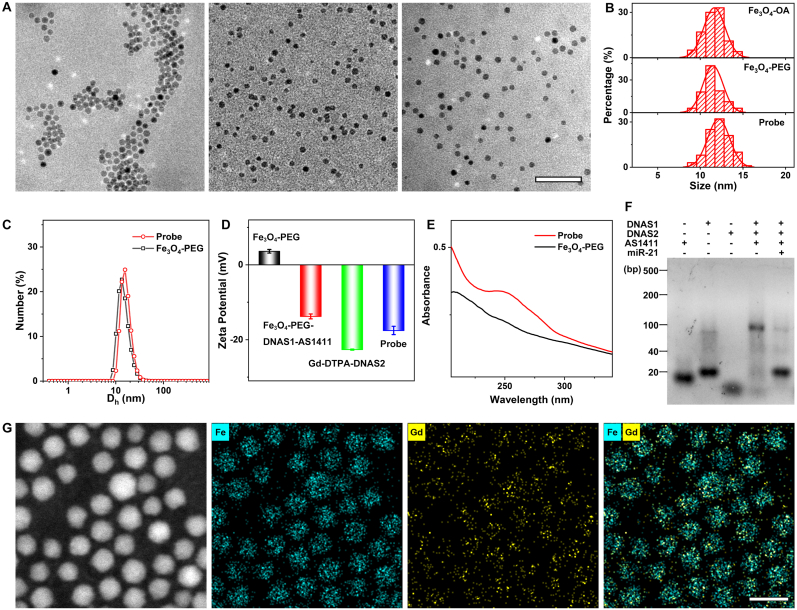
Characterization of nanoprobes. (A) TEM images of Fe_3_O_4_-OA, Fe_3_O_4_-PEG and nanoprobes. The embedded scale bar corresponded to 100 nm. (B) The histogram of the size of the Fe_3_O_4_-OA, Fe_3_O_4_-PEG and nanoprobes. (C) DLS results of Fe_3_O_4_-PEG and the nanoprobes. (D) The zeta potential of Fe_3_O_4_-PEG, Fe_3_O_4_-PEG-DNAS1-AS1411, Gd-DTPA-DNAS2 and the nanoprobes in water, respectively. (E) The UV-Vis absorption spectra of Fe_3_O_4_-PEG and the nanoprobes. (F) Agarose gel (4%) electrophoresis image of DNA assembly. (G) The energy dispersive spectrometry (EDS) result of the nanoprobe. The embedded scale bar corresponded to 20 nm.

The biocompatible miR-21-triggered nanoprobes with favorable performances were synthesized. Firstly, the core Fe_3_O_4_ nanoparticles were constructed. In detail, the oleate-capped Fe_3_O_4_ nanoparticles (Fe_3_O_4_-OA) were firstly fabricated by the high temperature thermal decomposition, with a uniform distribution at a diameter of 11.7 ± 1.3 nm, as shown in the transmission electron microscopy (TEM) image (Fig. 1A left and Fig. 1B top). Secondly, through the ligand exchange, the poly (ethylene glycol) (PEG) ligands bearing a maleimide group at one end and a diphosphate group at the other (dP-PEG-MAL) were applied to replace the oleate ligand, endowing the nanoparticles with hydrophilia and biocompatibility. The resultant water-soluble Fe_3_O_4_ nanoparticles (Fe_3_O_4_-PEG) have an average diameter of 11.6 ± 1.3 nm (Fig. 1A middle and Fig. 1B middle). Based on the click chemistry reaction between the maleimide group of Fe_3_O_4_- PEG and the sulfydryl at the end of DNA strands, the DNA single strand (DNAS1) can be effectively anchored to the surface of Fe_3_O_4_-PEG (Fe_3_O_4_-PEG-DNAS1) and the AS1411 aptamer were bonded to DNAS1(Fe_3_O_4_-PEG-DNAS1-AS1411). Subsequently, the other DNA single strand (DNAS2) linked with Gd-DTPA (Gd-DTPA-DNAS2) were bonded to DNAS1 through self-assembly procedures as well (Detailed information of all DNA sequences is listed in Table S1). Finally, the miR-21-triggered nanoprobes were obtained with an average diameter of 12.1 ± 1.3 nm (Fig. 1A right and Fig. 1B bottom). The selected area electron diffraction (SAED) of Fe_3_O_4_ nanoparticles and nanoprobes were investigated, and as displayed in Fig. S2, the lattice spacings of nanoparticles and nanoprobes match well, indicating that the spinel structure is preserved during preparation.

The aqueous dispersity of nanoprobes was tested through the dynamic light scattering (DLS) analysis. As given in Fig. 1C, both Fe_3_O_4_-PEG nanoparticles and nanoprobes exhibit a relatively narrow size distribution, and notably, the average hydrodynamic diameter (D_h_) of Fe_3_O_4_-PEG nanoparticles increased from 15.0 nm to 16.7 nm after linking with the corresponding DNA, indicating the successful synthesis of nanoprobes. Zeta potential measurements at each step of nanoprobes fabrication were investigated (Fig. 1D). In aqueous solution, the zeta potential of Fe_3_O_4_-PEG is 3.6 ± 0.5 mV, while due to the linkage of DNA, a negatively charged polymer [25], the zeta potential of Fe_3_O_4_-PEG-DNAS1-AS1411 transitioned to −13.8 ± 0.7 mV, and the zeta potential of nanoprobes decreased to −17.5 ± 1.1 mV, suggesting the successful DNA linkage. In addition, the UV-Vis absorption spectra of Fe_3_O_4_-PEG nanoparticles and nanoprobes were employed (Fig. 1E). This result reveals a prominent absorption peak at 260 nm in the nanoprobe solution after DNA modification, further confirming the successful DNA linkage. Furthermore, a standard curve for the combined DNA (DNAS1, DNAS2 and AS1411 aptamer) was presented in Fig. S3, and by using this curve, the surface-bound DNA concentration of nanoprobes was calculated to be 0.73 μM at an Fe concentration of 0.02 mg/mL. The designed DNA complementary base pairing strategy was assessed by using agarose gel electrophoresis (AGE). As displayed in Fig. 1F and Fig. S4, the AS1411 aptamer, DNAS1 and DNAS2 can effectively bind together. After adding miR-21 (which can bind to DNAS2, as verified in Fig. S5), the electrophoresis image shows significant changes in DNA pairing, confirming the feasibility of nanoprobes for miR-21 recognition. To validate the specificity of the DNA self-assembly strategy, the control RNA that cannot hybridize with DNAS2 was used (Fig. S6). The other miRNAs including miR-155 and miR-10b with the structure similar to miR-21 were chosen as well (Fig. S7). No discernible changes in the electrophoresis images were observed after mixing all the control RNAs with DNAS2. Therefore, the nanoprobe has specific activation for miR-21.

In addition to the investigation of the self-assembly strategy, the success of Gd-DTPA anchoring need to be assessed. The energy-dispersive spectroscopy (EDS) analysis with the Fe and Gd element was conducted. As shown in Fig. 1G, Gd elements is closely surrounding the Fe elements, indicating the successful linkage of Gd-DTPA. The TEM and TEM-EDS mapping data of the nanoprobe after co-incubation with miR-21 were provided (Fig. S8 and S9). The overall structural integrity and elemental types of the nanoprobe remain unchanged. However, the localization of Gd elements is shifted away from the Fe_3_O_4_ core. This is because miR-21-mediated competition leads to the release of some Gd-DTPA from the nanoprobe surface, altering the local distribution of paramagnetic Gd elements. In addition, FTIR spectrum of Fe_3_O_4_-PEG, Fe_3_O_4_-PEG-DNAS1-AS1411 and nanoprobes was used to further the success synthesis of nanoprobes (Fig. S10). The bands at ∼1070 cm^−1^ (C–O–C stretching vibration) and at ∼2875 cm^−1^ (C–H stretching vibration) confirmed PEG binding. DNA-specific PO_2_^−^ vibration at ∼1240 cm^−1^ and nucleobase bands at 1620-1680 cm^−1^ can be observed. In addition, the intensity inversion at 612/654 cm^−1^ and 900/942 cm^−1^, together with attenuation of DNA bands, indicated successful DNA hybridization and Gd-DTPA conjugation. Finally, the nanoprobes were characterized by ICP-MS, with a result that the Gd concentration is 0.14 mg/mL and Fe concentration is 3.08 mg/mL. All above results indicated that the successful fabrication of miR-21-triggered nanoprobes.

Furthermore, the stability of the nanoprobe in the environment of 10%FBS or pH 6.0 were evaluated as shown in Fig. S11. At different time points (0, 2, 4, 8, and 12 h) at 37 °C, the filtrates were collected via ultrafiltration, and the Gd element was quantified by ICP-MS. The results showed that the variation in Gd concentration was negligible over the entire incubation period, confirming the stability of the nanoprobe prior to tumor targeting and MRET response.

**Fig. 2:**
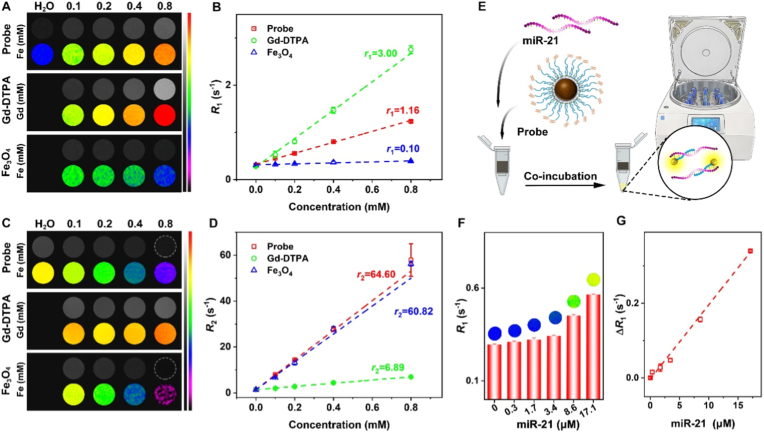
Relaxation property of the nanoprobes. *T*_1_ MR images (A) and *T*_2_ MR images (C) of nanoprobes, Fe_3_O_4_ nanoparticles and Gd-DTPA, respectively. The longitudinal relaxation rate (*R*_1_) (B) and the transverse relaxation rate (*R*_2_) (D) of nanoprobes, Fe_3_O_4_ nanoparticles and Gd-DTPA, respectively, together with the corresponding linear fittings for extracting the longitudinal and the transverse molar relaxivity (*r*_1_ and *r*_2_). (E) The schematic illustration of the filtrate approach to show the activated *T*_1_ signal. (F) *R*_1_ values of nanoprobes with different miR-21 additions. (G) The changed *R*_1_ (Δ*R*_1_) value of nanoprobes with different miR-21 concentration.

To assess the potential of nanoprobes as *T*_1_/*T*_2_ dual-modality MRI contrast agents, the relaxivity were measured by using a 7.0 T MRI instrument (Bruker BioSpec 70/20), as detailed in the experimental section. Firstly, the basic longitudinal relaxation rate (*R*_1_) andthe transverse relaxation rates (*R*_2_) of nanoprobes, and the corresponding *T*_1_-and *T*_2_-weighted images of nanoprobes were investigated with different Fe concentrations. The Fe_3_O_4_ nanoparticles and the commonly used clinical contrast agent Gd-DTPA were compared with the nanoprobes. As is well known, Gd-DTPA exhibits high *T*_1_ and low *T*_2_ profiles, primarily functioning as a *T*_1_ MRI contrast agent. In contrast, Fe_3_O_4_ nanoparticles show high *T*_2_ and low *T*_1_ characteristics, serving mainly as *T*_2_ MRI contrast agents. As shown in Fig. 2A and B, the *T*_1_-weighted images of the nanoprobe exhibits a concentration-dependent brightening effect, with signal intensity increasing gradually as the Fe content rises (*r*_1_ = 1.16 mM^−1^ s^−1^). However, the brightness of the nanoprobe remains significantly lower than that of Gd-DTPA (*r*_1_ = 3.00 mM^−1^ s^−1^), but higher than that of pure Fe_3_O_4_ nanoparticles (*r*_1_ = 0.10 mM^−1^ s^−1^). In Fig. 2C and D, the *T*_2_-weighted images reveals that the nanoprobe produces distinct negative contrast (*r*_2_ = 60.82 mM^−1^ s^−1^), with a magnitude comparable to that of pure Fe_3_O_4_ nanoparticles (*r*_2_ = 64.60 mM^−1^ s^−1^) but much higher than that of Gd-DTPA (*r*_2_ = 6.89 mM^−1^ s^−1^). In addition, the key intermediates Fe_3_O_4_-PEG-DNAS1-AS1411 (*r*_1_ = 0.11 mM^−1^s^−1^, *r*_2_ = 68.93 mM^−1^s^−1^) and Gd-DTPA-DNAS2 (*r*_1_ = 3.13 mM^−1^s^−1^, *r*_2_ = 6.04 mM^−1^s^−1^) were also evaluated (Fig. S12), showing comparable properties to pure Fe_3_O_4_ and Gd-DTPA, respectively. These results indicated that the nanoprobes possess high *r*_2_ and low *r*_1_. This means that the non-activated nanoprobes can serve as the usual superparamagnetic contrast agents. This is because that the superparamagnetic quencher Fe_3_O_4_ restricts the paramagnetic enhancer Gd-DTPA owing to the MRET effect [23]. The paramagnetic performance of Gd-DTPA becomes apparent when Gd-DTPA and Fe_3_O_4_ are separated by an appropriate distance [[26], [27], [28]].

In this work, the cancer-related miR-21 was utilized to drive the separation of Gd-DTPA and Fe_3_O_4_. The miR-21 can be perfectly hybridize with DNAS2 that carries Gd-DTPA, thereby moving paramagnetic Gd away from the interference of the superparamagnetic quencher, and triggering *T*_1_ signal activation. The signal activation schematic illustration was displayed as follows (Fig. 2E). The miR-21 were added to the nanoprobe solution, and the mixture was shaken for 2 h. The mixture was then ultrafiltered, and the filtrate was collected for further analysis. The *R*_1_ values together with the corresponding *T*_1_-weighted images at different miR-21 addition were acquired as shown in Fig. 2F. As miR-21 addition increased, *T*_1_ signals enhanced, and the *R*_1_ values increased accordingly. In brief, miR-21 content was closely correlated with the changed *T*_1_ signal, that is, the changed *R*_1_ (Δ*R*_1_). The relationship between Δ*R*_1_ and miR-21 concentration was summarized: when miR-21 concentration increased from 0.3 to 17.1 μM, Δ*R*_1_ increased linearly (Fig. 2F). The limit of detection (LOD) for miR-21 was determined to be 0.69 μM. These results validate the feasibility of MRET strategy and emphasize the potential capacity of nanoprobes for precisely detecting and quantitatively analyzing of the tumor-related miR-21. Apart from the *R*_1_ signal change in filtrate, the *R*_1_ (with *T*_1_-weighted images) and *R*_2_ values of nanoprobes are provided, which directly shows the variation in *R*_1_ and *R*_2_ with different miR-21 concentration. As illustrated in Fig. S13 and S14, the *R*_1_ values and corresponding *T*_1_-weighted images change progressively with increasing miR-21 concentration corresponding to the aforementioned results, whereas the *R*_2_ values remain largely unchanged. To further characterize this response, the *R*_1_ and *R*_2_ values were measured over time at a fixed miR-21 concentration (17.1 μM). As illustrated in Fig. S13 and S15, the *R*_1_ values and the corresponding *T*_1_-weighted images exhibit time-dependent changes until 4 h, whereas the *R*_2_ values remain largely unchanged as well.

### 2.2 Biosafety assessment of nanoprobes

**Fig. 3:**
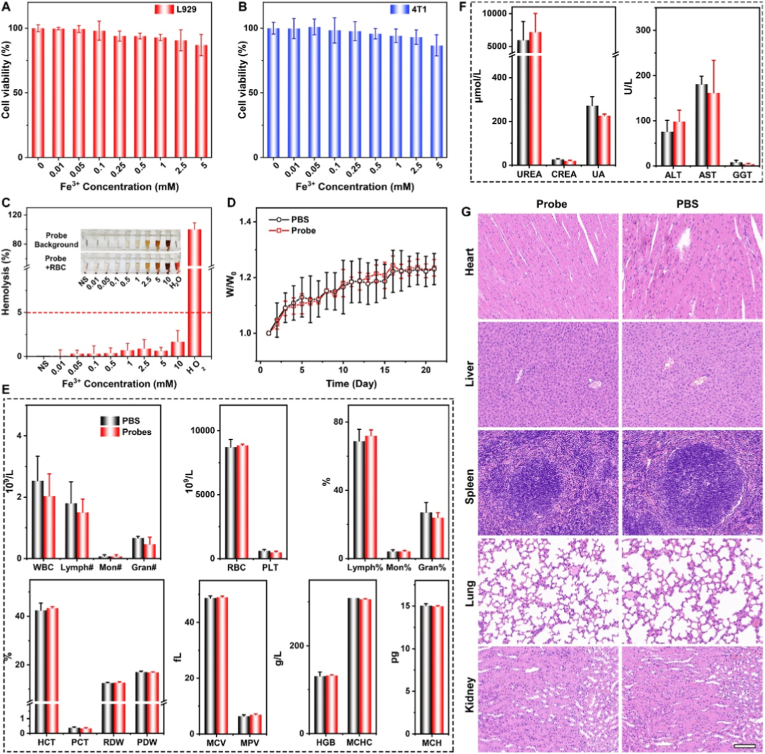
Biosafety assessment of nanoprobes. Viabilities of L929 cells (A) and 4T1 cells (B) treated with nanoprobes with different Fe concentrations. (C) Hemolysis rate of nanoprobes with different Fe concentrations. Inset: the photographs of nanoprobe solutions with different Fe concentrations with or without red blood cells (RBCs) after centrifugation. Body weight (n = 3) (D) and blood test (E)-(F) of healthy mice and nanoprobes-treated mice (n = 3). (G) H&E staining of the main organs from of healthy mice and nanoprobes-treated mice. The embedded scale bar corresponded to 100 μm. Abbreviations: WBC, white blood cell; Lymph, lymphocyte; Mon, monocyte count; Gran, granulocyte; RBC, red blood cell; PLT, platelet; MCV, mean corpuscular volume; MPV, mean platelet volume; MCH, mean corpuscular hemoglobin; HCT, hematocrit; PCT, plateletcrit; RDW, red blood cell distribution width; PDW, platelet distribution width; HGB, hemoglobin; MCHC, mean corpuscular hemoglobin concentration; UREA, urea; CREA, creatinine; UA, uric acid; ALT, alanine aminotransferase; AST, aspartate aminotransferase; GGT, gamma-glutamyl transpeptidase.

The biosafety is an essential prerequisite for *in vivo* application. At first, the cytotoxicity of nanoprobes was evaluated through Cell Counting Kit-8 (CCK-8) cell proliferation assay on the mouse fibroblast cell line L929 and the mouse breast cancer cell line 4T1. As shown in Fig. 3A and B, no obvious toxicity appears in not only L929 cells but also 4T1 cells, and over 85% of cells were viable even when the Fe^3+^ concentration reached 5 mM that is greatly higher than the immediate blood concentration of nanoprobe (∼2.48 mM with respect to Fe) after intravenous injection (the circulating blood volume of ∼72 mL/kg in mice [29,30]), indicating the excellent biosafety of nanoprobes. In addition, the hemolysis test of nanoprobes was performed to verify the *in vitro* biosafety of nanoprobes. The water serves as the positive control and the normal saline (NS) serve as the negative control, corresponding to 100% and 0% hemolysis rate, respectively. As shown in Fig. 3C, the hemolysis rate exhibits the concentration-dependence with the Fe concentration varying from the 0.01 mM to 10 mM, and even as the Fe^3+^ concentration reached to 10 mM that is over 4-folds higher than the immediate blood concentration of nanoprobe, the hemolysis rate is still under 3%. This result highly meets the requirements of hemolysis threshold of 5% set by the International Organization for Standardization (ISO) and the American Society for Testing and Materials (ASTM) [31]. All the results demonstrated that the excellent biocompatibility of nanoprobes. Besides the *in vitro* assessments, the *in vivo* biosafety of nanoprobes for long time was further investigated.

To further assess the biosafety *in vivo*, six mice were randomly allocated to two groups (n = 3) and all were intravenously injected through the tail vein. One group was injected with nanoprobes (0.1 mmol/kg Fe) and the other injected with PBS (with the same volume) as the control. Whereafter, the body weight of the nanoprobes-treated mice and the control mice were monitored and recorded every day. After 21 days monitoring, all the mice were sacrificed, and the blood samples and the main organs (heart, liver, spleen, lung and kidney) were collected as well. As shown in Fig. 3D, the body weight of the nanoprobes-treated mice preformed a steadily rising trend with no significant difference from the control mice, indicating that no physiological damage generated to the mice during the nanoprobe metabolic process. The blood tests can confirm the biosafety as well. As shown in Fig. 3E and F, there are no obvious differences in any of the index between the two groups, and all the measured parameters including the blood routine and the liver and kidney function indicators remained in the normal ranges. In addition, the H&E staining (Fig. 3G) of the main organs was evaluated as well and no obvious changes in the pathological histology or cellular structure between the nanoprobes-treated mice and the control mice as well. These results reveal that nanoprobes would not make a negative influence on the mice health, suggesting that the administered dose level of nanoprobes for mice is secured.

To clarify the *in vivo* metabolic pathways of the nanoprobe, the healthy mice were sacrificed prior to administration and at different post-administration time points (10 min, 4 h, and 24 h). Liver, spleen, and kidney tissues were collected for histological analysis, including Prussian blue staining (to detect Fe) and Chlorophosphonazo (CPN) III staining (to detect Gd) as shown in Figs. S16 and S17. In Prussian blue staining, the uptake of few nanoprobes was observed in the liver at 10 min, with accumulated blue areas in the liver and spleen at 4 h, indicating Fe metabolism. At 24 h, only a small fraction of Fe remained in the liver and spleen. For CPN III staining, Gd was predominantly detected in the liver and spleen at 4 h, as similar as the Fe metabolism. Collectively, the liver and spleen emerged as the primary organs responsible for the metabolism of the nanoprobe components.

### 2.3 Targeting ability of nanoprobes

**Fig. 4:**
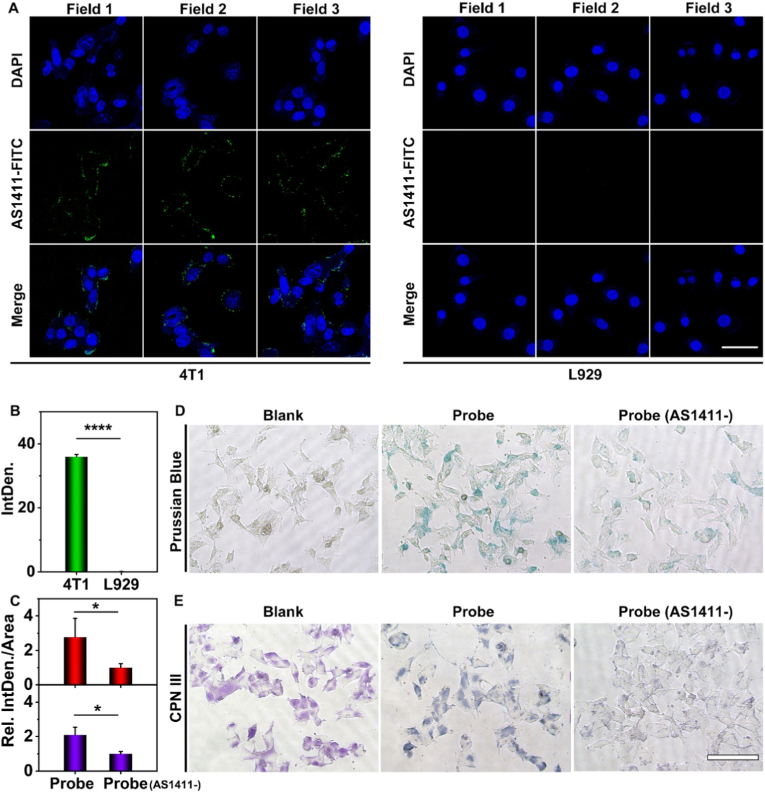
Targeting ability of nanoprobes. (A) Three fields of FITC-labeled AS1411 aptamer target 4T1 cells and L929 cells under confocal microscopy. Scale bar: 25 μm. (B) Quantitative analysis of the fluorescence integrated density of (A). (C) Quantitative analysis of the relative ratio of integrated density to area in 4T1 cells following co-incubation with the nanoprobe or AS1411-deficient probe (probe (AS1411^-^)) (Red: Prussian blue staining; Purple: Chlorophosphonazo (CPN) III staining). (D) The coincubation pictures with the nanoprobes or probes (AS1411^-^) stained by Prussian blue staining. (E) The coincubation with the nanoprobes or probe (AS1411^-^) stained by CPN III. Scale bar: 100 μm. Data are shown as mean ± SD. Statistical significance was determined by unpaired T-test (∗p < 0.05, ∗∗p < 0.01, ∗∗∗p < 0.001, ∗∗∗∗p < 0.0001).

To precisely detect and quantify the miR-21, it is crucial for the nanoprobes to effectively identify and enter the tumor cells after administration. It was reported that nucleolin is a membrane receptor overexpressed in breast cancer cells (4T1 cells), and AS1411 aptamer has a specific binding ability with nucleolin [26,[32], [33], [34]]. On this basis, 4T1 cells were chosen as the experimental group and L929 cells were used as the control. These cells were then employed in cell binding assays to assess the tumor-targeting capacity of nanoprobes. At first, the fluorescein isothiocyanate (FITC)-labeled AS1411 aptamer was prepared, and co-incubated with the two types of cells for 2 h, respectively. Subsequently, the 4′,6-diamidino-2-phenylindole (DAPI) staining was performed to label the cell nuclei. Finally, the laser scanning confocal microscopy (LSCM) was employed to visualize the targeting results. Three different fields of vision of 4T1 cells and L929 cells were selected for observation. As shown in Fig. 4A, obvious fluorescence signals are clearly observed on the surface of 4T1 cells, whereas negligible fluorescence signals are detected on L929 cells. Quantitative analysis revealed that the two cell types maintained comparable densities (Fig. S18), yet exhibited significantly divergent fluorescence intensities (p < 0.0001, 95% confidence interval, 34.67 to 37.02) (Fig. 4B). This result indicates that under identical cell density conditions, the AS1411 aptamer binds effectively to 4T1 cells but not to L929 cells. Collectively, these findings confirm the capacity of the AS1411 aptamer for specific targeting of malignant cells. In addition, the AS1411 aptamer facilitates the internalization of nanoprobes into cancer cells, enables nuclear targeting for miR-21. To verify this, nanoprobes modified with FITC-labeled AS1411 aptamer were co-incubated with cancer cells for 2 h. As shown in Fig. S19, green fluorescence from the FITC-labeled nanoprobes exhibited low co-localization with lysosomes (labeled with LysoTracker Red), indicating that the AS1411 aptamer mediates intracellular delivery of a portion of nanoparticles into cancer cells and targets the cell nucleus, as previously reported [35].

Tumor-targeting capacity of nanoprobes were assessed with Prussian blue staining and CPN III staining. At first, 4T1 cells and L929 cells were co-incubated with the nanoprobes for 6 h each. Following this, Prussian blue staining and CPN III staining were performed respectively, to allow the presence of Fe and Gd elements to be visualized and confirmed under a microscope, respectively [29,36]. The cells that are not treated with nanoprobes appeared colorless following Prussian blue staining, whereas they exhibited in purple after CPN III staining. Following co-incubation with nanoprobes, there was a noticeable alteration in the staining of the cells, with Fe elements showing up as blue (Fig. 4D) and Gd elements as dark green (Fig. 4E). In contrast, nanoprobes lacking the AS1411 aptamer (Probe (AS1411-)) exhibited significantly reduced blue signals or light purple in 4T1 cells. Quantitative analysis of these staining signals is presented, and normalization processing was carried out for the ratio of signal integrated density and cell density, as shown in Fig. 4C and Fig. S20. Nanoprobes possess a higher targeting capability toward 4T1 cells than the Probe (AS1411-) (p < 0.05, 95% confidence interval, up: 0.01269 to 3.546; down: 0.3272 to 1.748). This result indicates efficient uptake of nanoprobes by 4T1 cells. Additionally, staining analysis was performed to evaluate the uptake of nanoprobes by L929 cells (Fig. S21), and no discernible changes in staining signals were observed, further verifying the specific targeting ability of the nanoprobes for tumor cells.

### 2.4 Positional imaging of tumor

MiR-21 plays a significant role in the oncogenic process of cancer, which includes increased proliferation, invasion, and meta-static and decrease apoptosis [37]. Numerous malignancies commonly have high expression of miR-21 and the comparison of miR-21 expression of various cancer types from the OncoMir Cancer Database [38] was given in Fig. S22. On the whole, the top ten cancers are head and neck squamous cell carcinoma (HNSC), cervical squamous cell carcinoma and endocervical adenocarcinoma (CESC), lung squamous cell carcinoma (LUSC), lung adenocarcinoma (LUAD), esophageal carcinoma (ESCA), stomach adenocarcinoma (STAD), bladder urothelial carcinoma (BLCA), mesothelioma (MESO), colon adenocarcinoma (COAD), and breast invasive carcinoma (BRCA). Demonstrably, there was minimal variation in the high levels of miR-21 expression between the different types of cancer. In addition, the miR-21 expression between the tumor issues and the normal tissue is counted as shown in Fig. S23, deriving from the online resource OncomiR [39,40]. Notably, miR-21 expression exhibits a significant difference between BRCA tissues and healthy tissues. In detail, the expression level of miR-21 in BRCA tissues is 4.6-fold higher than that in healthy tissues. Furthermore, the upregulation of cancer-related miR-21 has been confirmed in the murine breast cancer 4T1 cell line as well [38]. Therefore, miR-21 is a crucial marker in the development of tumors; thus, the timely localization and quantification of miR-21 are conducive to enabling effective monitoring of tumor progression.

**Fig. 5:**
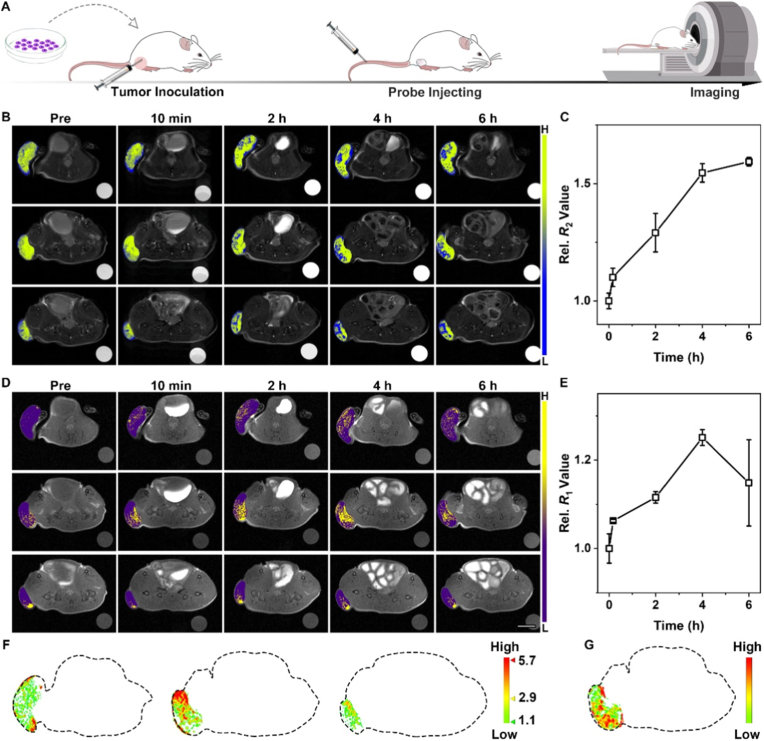
Positional tumor imaging. (A) Schematic diagram of tumor establishment and imaging. (B) The *T*_2_-weighted MR images of the subcutaneous tumor sections acquired before treatment and at different time points post-injection of nanoprobes. (C) The corresponding relative *R*_2_ value summarized at different time. (D) The *T*_1_-weighted MR images of the subcutaneous tumor sections acquired before treatment and at different time points post-injection of nanoprobes. Scale bar: 0.5 cm. (E) The corresponding relative *R*_1_ value summarized at different time. (F) Quantitative visualization of miR-21 *in vivo* with three planes obtained at 4 h post-injection in tumor. (G) The distribution of *T*_2_ signal intensity obtained at 4 h post-injection in tumor (Corresponding with the second slice).

To further assess the tumor-targeting and miR-21 imaging abilities of nanoprobes *in vivo*, 4T1 subcutaneous tumor models were established in BALB/c mice. After 7 days inoculation, BALB/c mice were intravenously injected with nanoprobes and were investigated on the 7.0 T MRI scanner. The schematic diagram of tumor establishment and imaging procedures is shown in Fig. 5A. The MR images including *T*_1_-and *T*_2_-weighted MR images of the tumor acquired before the administration and at different time points post-injection of nanoprobes are shown in Fig. 5B and D. In the MRI images, blue denotes the negative *T*_2_ signals, and yellow denotes the positive *T*_1_ signals. As shown in Fig. 5B, no obvious *T*_2_ signals were observed at the tumor site prior to intravenous administration of the nanoprobes. In contrast, 10 min after injection of the nanoprobes (at a Fe concentration of 0.1 mmol/kg), the nanoprobes gradually accumulated at the tumor site due to the tumor-targeting ability of the AS1411 aptamer, finally resulting in the increasingly enhancement of *T*_2_ signals. Importantly, the *T*_2_ signals are progressively increasing for 6 h across all the three different tumor planes. This observation not only confirms the excellent targeting capability of the nanoprobes but also indicates their prolonged blood circulation time, which provides sufficient duration for effective tumor visualization. The corresponding relative *T*_2_ values were quantified to further characterize nanoprobe accumulation (Fig. 5C). At 4 h post-injection, the relative *T*_2_ value of mice treated with the nanoprobes exhibited a 1.55-fold increase compared to the signals at the pre-injection. By 6 h post-injection, the relative *T*_2_ value continued to rise, reaching a 1.59-fold increase relative to the pre-injection level. This trend in relative *T*_2_ values is consistent with the sustained enhancement of *T*_2_ signals observed in the MRI images.

The *T*_1_ images exhibited a distinctly different signal trend compared to the *T*_2_ images. As shown in Fig. 5D, following nanoprobe administration, the *T*_1_ signals were notably triggered. This was attributed to the immediate interaction between the accumulated nanoprobes and the excessive miR-21 present at the tumor site. Specifically, as the nanoprobes targeted and accumulated in 4T1 tumor cells, DNAS2 on the nanoprobe surface recognized and hybridized with the overexpressed miR-21 in the tumor cells. This hybridization event drove the separation of Gd-DTPA from Fe_3_O_4_ nanoparticles, thereby activating strong *T*_1_ signals. The distribution and intensity of *T*_1_ signals possess a strong correlation with the location and concentration of miR-21 in tumor cells. Over time, *T*_1_ signals remained triggered but reached their maximum intensity at 4 h post-injection. At 6 h post-injection, the *T*_1_ signals had decreased. This signal decline may be ascribed to the more rapid metabolism of Gd-DTPA released from the nanoprobes compared to that of Fe_3_O_4_ nanoparticles.

Statistical analysis of relative *T*_1_ signals over the 6 h post-injection period (Fig. 5E) showed as the follows: at 4 h post-injection, the relative *T*_1_ signal of the nanoprobe-treated group was 1.25-fold higher than the initial *T*_1_ value. However, at 6 h post-injection, the relative *T*_1_ signal had declined. Apart from the rapid metabolism of Gd-DTPA, the gradual accumulation of Fe_3_O_4_ nanoparticles may potentially affect the *T*_1_ signal as well. The variation trend of the relative *T*_1_ signals was consistent with that observed in the MR images. In addition, the distribution of *T*_1_ and *T*_2_ signals within the tumor was found to be not completely overlapping and this observation was further verified via image processing. Collectively, these results demonstrate the potential of the nanoprobes for accurate tumor localization and miR-21 detection.

### 2.5 Quantitative analysis of miR-21

The aforementioned results demonstrate that the *T*_1_ signals signal changes are due to the specific recognition of miR-21 of nanoprobes. Therefore, the quantization of miR-21 can be achieved by extracting the variation value of the *T*_1_ signal in the image. To avoid the interference of pre-injection images on post-injection images, the *T*_1_ images reflecting signal changes can be obtained via subtraction calculation of these two sets of images (pre-injection vs. post-injection). The miR-21 distribution map can be obtained by performing image processing operations on the images acquired at 4 h post-injection. Benefiting from the *in vitro* quantification of miR-21with Δ*R*_1_, quantitative visualization of this cancer-associated miRNA *in vivo* is feasible when coupled with the distinct Δ*R*_1_ values of MR images at 4 h post-injection, as shown in Fig. 5F. MiR-21 exhibits an uneven distribution within the tumor. Specifically, the miR-21 concentration distribution map (color gradient from green to yellow to red indicating low to high concentrations) clearly reflects the spatial heterogeneity of miR-21 expression within the tumor (the second plane as an example), with the highest concentration (5.7 μM) in the top-right region and relatively low levels in the bottom-right region. Therefore, the localization and quantification of tumor-associated RNAs can be effectively realized through this strategy.

**Fig. 6:**
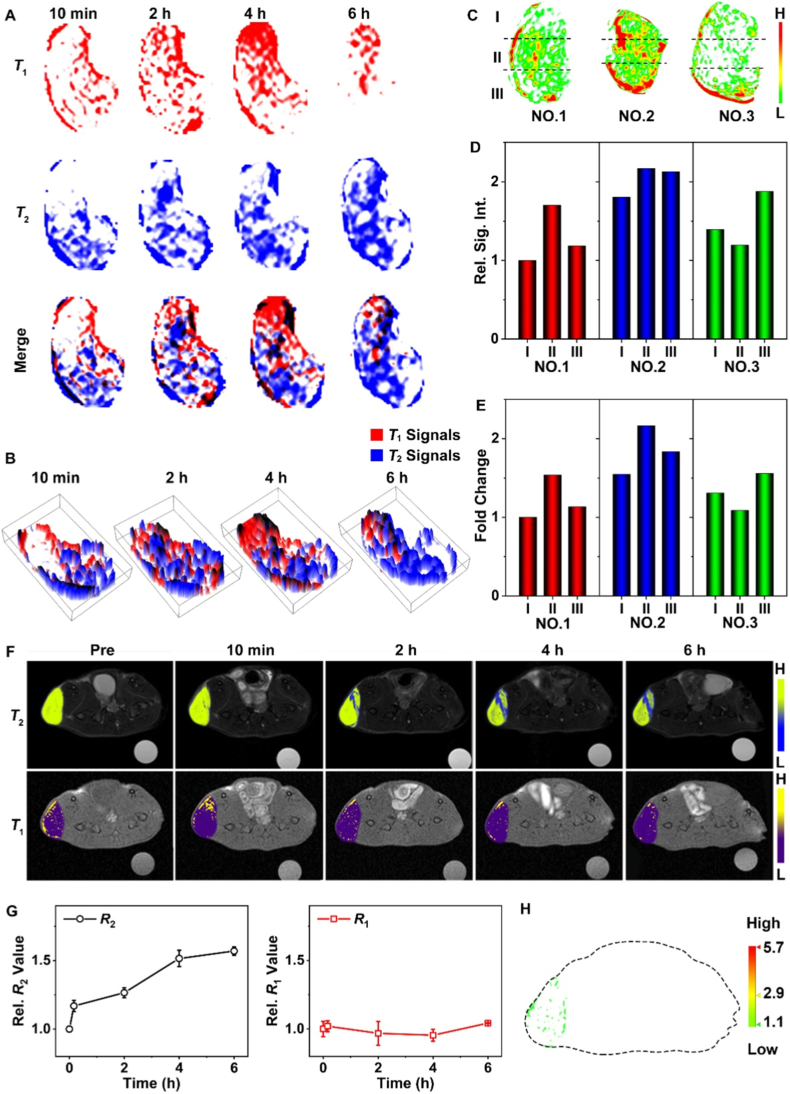
Quantitative analysis of tumor imaging. (A) The distribution of *T*_1_ signals (Red), the *T*_2_ -signals (Blue) and the merge images for the second plane in Fig. 5F. (B) 3D stereograph of the merge distribution of *T*_1_ and *T*_2_ signals at different time. (C) MiR-21 distribution in tumor tissues from three independent mice at 4 h post-injection, with each tumor sectioned into three distinct regions. (D) Average signal intensity of each corresponding tumor region. (E) Fold change of each corresponding tumor region determined by qRT-PCR. (F) The *T*_1_ - and the *T*_2_ -weighted MR images of the 4T1 subcutaneous tumor sections acquired before injection and at different time points post-injection of the control nanoparticles. (G) The corresponding relative relaxation rate of *T*_1_ - and *T*_2_ -weighted MR images by using control nanoparticles. (H) The quantitative miR-21 visualization after injecting the control nanoparticles *in vivo*.

Furthermore, based on this image-based calculation method, the accumulation and distribution of *T*_2_ signals (the second plane) at 4 h post-injection were obtained as shown in Fig. 5G. The *T*_2_ signal intensity was throughout the entire tumor, with the highest local concentration of aggregated nanoprobes observed in the lower-left part of the tumor. Notably, the distribution of high-intensity *T*_1_ and *T*_2_ signals is not completely overlapping. To investigate the time-dependent differences in the distribution of *T*_1_ and *T*_2_ signals, the enhanced *T*_1_-and *T*_2_-weighted images were merged after eliminated removing pre-injection background interference from each time point (Fig. 6A). The distribution relationship between the two signals can be observed more clearly via a 3D stereograph. As shown in Fig. 6B, the distribution of *T*_1_ and *T*_2_ signals exhibits a bit difference at all varying time points. The significant heterogeneity of malignant tumors serves as one of the contributing factors to this mismatched distribution of *T*_1_ and *T*_2_ signals. To further confirm the reliability and reproducibility of MRI-based miR-21 quantification strategy and verify the universality of intratumoral miR-21 heterogeneity, the additional validation experiments were conducted using three independent mice. Specifically, the three mice were euthanized immediately after imaging at the 4 h time point, with the tumor tissue completely excised and sectioned into three regions, roughly following the dotted line in Fig. 6C. The fold change of miR-21 in these specific regions was tested by qRT-PCR, and the corresponding relative signal intensity of each region was also contrastively analyzed. The qRT-PCR results of miR-21 signals in various regions show obvious distribution differences, further confirming the heterogeneous expression of miR-21 within individual tumors. As shown in Fig. 6D and E, the miR-21 content change in each region showed a strong correlation with the intensity change in MRI results.

In addition, to further validate the feasibility of the strategy for miR-21 targeting and quantification, control nanoparticles were prepared. The control nanoparticles were synthesized, but modified with a mis-matched DNA sequence (DNAS3). Specifically, DNAS3 cannot hybridize with miR-21, so these control nanoparticles cannot trigger *T*_1_ signal activation. After intravenous injection, as shown in Fig. 6F, the *T*_2_ signals at the tumor site were enhanced to a similar extent as those in the nanoprobe-treated group, while no significant change of *T*_1_ signal was detected. Furthermore, quantitative analysis (Fig. 6G) demonstrated that the relative *T*_2_ values of the control group showed a 1.52-fold increase at 4 h post-injection and a 1.57-fold increase at 6 h post-injection, respectively, as similar as the nanoprobe-treated group. In contrast, the relative *T*_1_ values of the control group remained almost unchanged throughout the entire observation period. Image processing of the *T*_1_-weighted images confirmed this result as well (Fig. 6H). All results demonstrated that the resultant nanoprobes can accurately target the tumor, and further localize and quantify miR-21. In addition, the CT26 tumor model (a commonly used colon cancer model with endogenous miR-21 expression) was performed to further verify the *in vivo* MRI experiments. As shown in Fig. S24, the CT26 tumors also exhibited significant miR-21-triggered *T*_1_ signal enhancement, which is consistent with the results obtained from the 4T1 tumor model. This result confirmed the universality of nanoprobe specifically responding to endogenous miR-21.

## 3 Conclusions

A *T*_1_/*T*_2_ dual-modality MRI nanoprobe stemming from MRET was constructed for *in vivo* oncogene position and quantification. Due to the specific target of the AS1411 aptamer to nucleolin, the nanoprobes can accurately identify tumor cells, and owing to MRET effect, the nanoprobe can localize cancer-associated RNA without the interference of background.

It was reported that the previous MRET-based MRI nanoprobes utilizing Fe_3_O_4_ exhibit excellent performance in tumor identification as well, including distinguishing inflammatory regions and tumor [27,41,42], monitoring tumor growth with telomerase activity [26] or detecting miRNAs *in vivo* [28]. These works verify the feasibility of Fe_3_O_4_/DNA/Gd chelates-based MRET effects for tumor imaging, but their ability to quantitatively analyze tumor-related biomarkers remains limited. In contrast, benefiting from the *in vitro* quantification of miR-21with Δ*R*_1_, our work can quantitatively visualize miR-21 *in vivo*, holding great potential for precise MRI and early cancer diagnosis. The nanoprobes exhibit a LOD of 0.69 μM for miR-21, and the AS1411 aptamer modification efficient targeted delivery to miR-21-highly-expressing tumor cells, including both 4T1 and CT26 cells. In addition, the distribution of *T*_1_ and *T*_2_ signals at various periods in one image were displayed, suggesting the spatial heterogeneity of tumors. Furthermore, the *in vivo* distribution of miR-21 visualized by the nanoprobes is consistent with the compared results of qRT-PCR with different tumor site, confirming the reliable quantitative accuracy of our strategy.

Overall, the miR-21-triggered nanoprobes that can timely and precisely evaluate the change of the cancer-associated RNA were developed, offering an effective strategy for early diagnosis of tumors.

## 4 Experimental section

### 4.1 Synthesis of iron oleate

Firstly, to synthesize the iron oleate, 120 mmol NaOH, 60 mL water, 80 mL ethanol, and 120 mL oleic acid (OA) were mixed firstly and then, 40 mmol FeCl_3_⋅6H_2_O and 140 mL cyclohexane were added, agitating at 70 °C for about 4 h. The resulting materials were washed with hot water for several times to remove other remnants, and could be finally obtained by rotary evaporation.

### 4.2 Synthesis of Fe_3_O_4_-OA

4 mmol obtained iron oleate, 4 mmol oleic acid and 25 mL 1-octadecene were mixed. Then, the mixture was heated to 150 °C under a vacuum and then heated to 310 °C at a rate of 3.3 °C min^−1^ and maintained at 310 °C for 30 min under nitrogen protection. The resulting solution was cooled to the room temperature and was precipitated and washed by using ethanol with several times, and finally collected by magnetic separation. The resulting Fe_3_O_4_-OA was redispersed in cyclohexane for further experiments.

### 4.3 Synthesis of Fe_3_O_4_-PEG

Typically, 100 mg dP-PEG-MAL was dissolved in 15 mL THF and 10 mg Fe_3_O_4_ -PEG was dissolved in 5 mL of THF. Then, the above two solutions were mixed and reacted at 40 °C under stirring overnight. After that, the resulting nanoparticles were precipitated and washed with cyclohexane for three times, and then fully dried under vacuum at room temperature. Finally, the nanoparticles were dissolved by 4 mL Milli-Q water and were purified by ultrafiltration for 4 cycles by using a 30 kDa MWCO centrifugal filter (Millipore YM-30) at 2000 g in order to remove excess PEG ligand.

### 4.4 Synthesis of nanoprobes

Before synthesis, all the used DNA sequences were dissolved in the TAE-Mg^2+^ buffer (40 mM Tris buffer, 2 mM EDTA, and 12.5 mM Mg (CH_3_COO)_2_) with the concentration of 25 μM, quickly heated to 95 °C for 5 min and then cooled to room temperature. (1) Preparation of Fe_3_O_4_-PEG-DNAS1-AS1411. Fe_3_O_4_-PEG (containing 0.5 mg Fe^3+^), 12.3 nmol AS1411 aptamer and 12.3 nmol DNAS1 were mixed and reacted for 6 h at 37 °C to obtain the Fe_3_O_4_-PEG-DNAS-AS1411 aptamer (final volume: 1.2 mL). DNAS1 sequence linked with a thiol group can be anchored by the maleimide residue on the surface of nanoparticles, through the click reaction. AS1411 aptamer can linked with DNAS1 through the complementary base pairing. (2) Preparation of DNAS2-Gd. 12.1 mg DTPA was dissolved in 1 mL TAE-Mg^2+^ buffer and adjust the pH (=8.5) by using 0.1 M NaOH. Then the DMTMM (2.3 mg dissolved in 0.5 mL 0.1 M NaOH water solution) was added quickly and the mixture was stirred for 10 min at room temperature. After that, the 150 μL aforementioned solution and 12.3 nmol DNAS2 were combined, with the final volume of 1.2 mL, and the mixture was stirred for 4 h at 37 °C. Next, the obtained DNAS2-DTPA was purified by ultrafiltration to remove the free DTPA via TAE-Mg^2+^ buffer and 3k MWCO centrifugal tube. After that, 5 mg GdCl_3_·6H_2_O was dissolved in 1 mL TAE-Mg^2+^ buffer with a pH = 4 and then 150 μL of the resulting Gd^3+^-containing solution was added slowly into DNAS2-DTPA solution, stirring for 2 h at 37 °C. The obtained DNAS2-Gd was purified by ultrafiltration to remove the free Gd^3+^ via TAE-Mg^2+^ buffer and 3k MWCO centrifugal tube. (3) Preparation of nanoprobes. The aforementioned resulting solution including the Fe_3_O_4_-PEG-DNAS1- AS1411 aptamer and DNAS2-Gd were mixed for another 2 h at 37 °C and finally the nanoprobes are obtained after purified and concentrated by ultrafiltration with the 30 k MWCO centrifugal tube.

### 4.5 Relaxivity measurements

The relaxivity measurements were carried out on a 7.0 T MRI scanner. The aqueous solutions of nanoprobes with different Fe^3+^ concentration and the filtrates of Gd^3+^-containing solution were prepared in 200 μL Eppendorf tubes.

The *T*_1_ and *T*_2_ values were measured through *T*_1_map and *T*_2_map sequences. The parameters of *T*_1_map were set as follows: echo time (TE) = 5.90 ms, repetition time (TR) = 3000, 1500, 1000, 500, 298 ms, field of view (FoV) = 35 mm × 35 mm, slice thickness = 1 mm. The parameters of *T*_2_map were set as follows: TE = 6.6, 13.2, 19.8, 26.4, 33, 39.6, 46.2, 52.8, 59.4, 66, 72.6, 79.2 ms, TR = 3000 ms, FoV = 35 mm × 35 mm, slice thickness = 1 mm.

The *T*_1_ and *T*_2_ images were measured through *T*_1_-weighted imaging and *T*_2_-weighted imaging. The parameters of *T*_1_-weighted imaging were set as follows: TR = 300 ms, TE = 5.01 ms, FoV = 35 × 35 mm, MTX = 200 × 200, slice thickness = 1 mm. The parameters of *T*_2_-weighted imaging were set as follows: TR = 3000 ms, TE = 40 ms, FoV = 35 × 35 mm, MTX = 200 × 200, slice thickness = 1 mm.

### 4.6 ΔR and the limit of detection of miR-21 *in vitro*

ΔR was obtained by subtracting the value of the original value with miR-21 concentration of 0 μM, and a linear fitting was performed to calculate the slope. Then, according to the equation: LOD = 3σ/S (σ is the standard deviation and S is the slope of the liner curve), the limit of detection for miR-21 can be determined.

### 4.7 In vivo MRI studies

The detailed parameters for *T*_1_-weighted imaging of tumor models with the body coil were set as follows: TE = 5.08 ms, and TR = 389.53 ms, FoV = 35 mm × 35 mm, MTX = 200 × 200, slice thickness = 1 mm.

The detailed parameters for *T*_2_-weighted imaging of tumor models with the body coil were set as follows: TE = 40.0 ms, and TR = 3000.0 ms, FoV = 35 mm × 35 mm, MTX = 200 × 200, slice thickness = 1 mm.

### 4.8 Analysis of the relative T_1_ signal changes of tumors and quantification of miR-21

To analyze the relative *T*_1_ signal changes in tumors and quantify miR-21, the tumor MR images acquired 4 h post-administration and before administration were performed with image operations using Image J. The corresponding *T*_1_ relaxation time values in the two images were extracted for measurements. The values were then correlated with the *in vitro* signal *ΔR* to determine the miR-21 concentration. Finally, according to corresponding *T*_1_ relaxation time and the calculated miR-21 concentration to draw the corresponding pseudo-color images.

### 4.9 Statistical analysis

Data are expressed as means ± SD as indicated in the figure captions. Statistical differences of two groups were determined by T test. All tests were carried out by GraphPad Prism software (v7.0 and v8.0), and the charts were drawn by OriginPro (9.0 and 2019b) and GraphPad Prism software (v8.0).

All animal experiments were performed according to a protocol approved by the China-Japan Friendship Hospital Institutional Animal Care and Use Committee (zryhyy61-21-03-27).

## CRediT authorship contribution statement

**Wenyue Li:** Data curation, Investigation, Methodology, Project administration, Writing – original draft, Writing – review & editing. **Runjie Wang:** Investigation, Methodology, Writing – review & editing. **Xinyi Zhang:** Investigation, Methodology, Writing – review & editing. **Shuai Wu:** Investigation, Methodology, Writing – review & editing. **Peisen Zhang:** Methodology, Writing – review & editing. **Hongxiang Feng:** Funding acquisition, Supervision, Writing – review & editing. **Yue Lan:** Project administration, Supervision, Writing – review & editing. **Zhuo Ao:** Conceptualization, Project administration, Supervision, Writing – review & editing. **Yi Hou:** Conceptualization, Funding acquisition, Project administration, Supervision, Writing – original draft, Writing – review & editing.

## Declaration of competing interest

The authors declare that they have no known competing financial interests or personal relationships that could have appeared to influence the work reported in this paper.

## Data Availability

All data needed to evaluate the conclusions in the paper are present in the paper and/or the Supplementary PDF.
