# Supplementary material for: Quantitative oncogene-mapping within malignant tumors through Multi-parameter MRI based on RNA-triggered nanoprobes
Source: Mater Today Bio. 2026 Feb 13;37:102936. doi: 10.1016/j.mtbio.2026.102936 (PMC12926584; doi:10.1016/j.mtbio.2026.102936)
Supplement: Multimedia component 1 [file mmc1.pdf]

Supplementary data

Quantitative Oncogene-mapping within Malignant Tumors through Multi-parameter MRI Based on RNA-triggered Nanoprobes

Wenyue Li[1,2,3,4], Runjie Wang[2], Xinyi Zhang[2], Shuai Wu[2,3], Peisen Zhang[1,2], Hongxiang Feng[4,], Yue Lan[1,]*, Zhuo Ao[3,]*, and Yi Hou[2,]**

[1]Department of Rehabilitation Medicine, School of Medicine, the Second Affiliated Hospital of South China University of Technology (Guangzhou First People's Hospital), Guangzhou, 510180, China.

[2]College of Materials Science and Engineering, and College of Life Science and Technology, Beijing University of Chemical Technology, Beijing 100029, China.

[3]CAS Center for Excellence in Nanoscience, National Center for Nanoscience and Technology, Beijing 100190, China.

[4]Department of Thoracic Surgery, China-Japan Friendship Hospital, Beijing 100029, China.

E-mail: houyi@iccas.ac.cn (Yi Hou); aoz@nanoctr.cn (Zhuo Ao); bluemooning@163.com (Yue Lan); fenghongxiang617@126.com (Hongxiang Feng)

Additional Experimental Section

Materials

Ferric trichloride hexahydrate ($FeCl_3 \cdot 6H_2O$, 99.0%) was purchased from Shandong Xiya Chemical Industry Company (Shandong, China). Oleic acid (OA), 1-octadecene (ODE) and $GdCl_3 \cdot 6H_2O$ were purchased from Sigma-Aldrich Co., Ltd (Shanghai, China). Cyclohexane was brought from Damao Chemical Reagent Co., Ltd., and anhydrous ethanol and tetrahydrofuran (THF) with analytical grade were brought from Sinopharm Chemical Reagent Beijing, Co., Ltd. Sodium hydroxide (NaOH) was obtained from Beijing Chemical Reagents Co. Ltd. The poly(ethylene glycol) (PEG) ligand (dP-PEG-MAL) with the molecular weight (PEG segment) of 2000 was provided by Beijing Oneder Hightech Co. Ltd. Magnesium acetate tetrahydrate, DNA Marker A (25~500 bp) and 4S Red Plus Nucleic Acid Stain (4S Red) was obtained from Sangon Biotech Co., Ltd. (Shanghai, China). 6× DNA loading buffer and 50× TAE electrophoresis buffer (2 mol/L Tris-acetate, 0.1 mol/L EDTA) was obtained from Wuhan Servicebio Technology Co., Ltd. Diethypyrocarbonate (DEPC) and the synthesis of DNA and RNA sequence were provided by QYAOBIO (ChinaPeptides Co., Ltd.). All sequences were HPLC-purified and lyophilized and were shown in Supporting Information Table S1. Lyso-Tracker Red was bought from Shanghai Beyotime Biotechnology Co., Ltd., China). The murine breast cancer 4T1 cell line (ATCC CRL-2539), the murine fibrobastic L929 cell line (ATCC CCL-1) and the mouse colon cancer CT26 cell line (CRL-2638) were purchased from American Type Culture Collection (ATCC).

Characterizations

Transmission electron microscope (TEM) images of the nanoparticles and nanoprobes were obtained on a JEM-2100 transmission electron microscope at an acceleration voltage of 200 kV. The core sizes of Fe_3O_4 nanoparticles and nanoprobes were measured by averaging at least 100 nanoparticles per sample in corresponding TEM image. Dynamic light scattering (DLS) was carried out at 298 K with Nano ZS

(Malvern) equipped with a solid state He-Ne laser (λ = 632.8 nm). The absorption of aqueous solution of nanoprobes was analyzed on the UV-Vis spectrophotometer (Thermo, MULTISKAN GO). The Fe and Gd concentrations in different systems were determined by inductively coupled plasma-mass spectrometry (ICP-MS, Thermo, ICAP-Qc).

Cell culture

4T1 cells and L929 cells were cultured in Dulbecco's Modified Eagle Medium (DMEM) with 10% FBS and 1% penicillin-streptomycin solution (100×) at 37 °C under a 5% CO_2 atmosphere in the cell incubator. CT26 cells were cultured in a RPMI-1640 medium with 10% FBS and 1% penicillin-streptomycin solution (100 ×) at 37 °C under a 5% CO_2 atmosphere in the cell incubator.

Cell viability assays

Cell Counting Kit-8 (CCK-8) assay on 4T1 cells and L929 cells was carried out as follows. Cells were seeded into a 96-well cell culture plate with a density of 5,000 cells per well under 100% humidity, and then cultured at 37 °C in an atmosphere containing 5% CO_2 for 24 h. Then, the nanoprobes with different concentrations were added into the wells and incubated with the cells for 24 h at 37 °C under 5% CO_2. After the supernatant containing the excrescent nanoprobes was decanted, the cells were incubated for another 48 h. After that, 10 μL CCK-8 was added to each well, and incubated for 4 h at 37 °C under 5% CO_2. The optical density of each well at 450 nm was recorded on a microplate reader (Thermo, MULTISKAN GO).

Hemolysis test

Briefly, 2 mL blood sample was mixed with 6 mL of normal saline (NS) to purify through the centrifugation for several times. The resultant red blood cells were diluted in 4 mL of NS and the suspension was then diluted 1: 4 into NS (negative control); water (positive control); nanoprobes (in NS) at different concentrations. The samples were kept in the dark for 4 h at 37 °C and then centrifuged at 3000 rpm for 5 min. After centrifuging, the supernatant was transferred to a 96-well plate and the absorbance at 541 nm was recorded.

The hemolysis rate was calculated by the following equation:

Hemolysis rate= $\frac{D_t - D_{nc}}{D_{pc} - D_{nc}} \times 100\%$

where D_t, D_{nc}, and D_{pc} were the absorbance of the tested sample, the negative control, and the positive control, respectively.

The specific target of AS1411 aptamer

4T1 cells and L929 cells were seeded into the confocal dishes at a density of 2.5×10^5 cells/dish under 100% humidity, and then cultured at 37 °C in an atmosphere containing 5% CO_2 overnight. Subsequently, the fluorescein isothiocyanate (FITC) marked AS1411 aptamers were diluted and added to confocal dishes. After 2 h co-incubation, the supernatant was aspirated and discarded, the cells were fixed by adding 4% paraformaldehyde. Finally, the nuclear dye the 4',6-diamidino-2-phenylindole (DAPI) was added and the luminescence images were captured on the laser scanning confocal microscopy (LSCM Leica TCS SP8). Approximately 10 cells in the field of view selected and the fluorescence intensity were statistically analyzed in three different fields of vision. All the quantitative data with the confidence intervals (95% CI).

Cell uptake of nanoprobes

The 4T1 cells and the L929 cells were used to perform the cell uptake experiments. Firstly, the 4T1 cells and L929 cells were seeded into a 24-well cell culture plate at a density of 4×10^4 cells/well under 100% humidity, and then cultured at 37 °C in an atmosphere containing 5% CO_2 overnight. Subsequently, after the supernatant was diluted and discarded, nanoprobes with the Fe^{3+} concentration of 100 mg/L attenuated by DMEM were added to the wells to co-incubate with the cells. After 6 h, the supernatant was aspirated and discarded, the cells were fixed by adding 4% paraformaldehyde. Finally, the cells were stained with Prussian blue and CPN III and the cells were observed by fluorescence microscope (Leica DMI 3000B). Approximately 50 cells per field of view were analyzed and three different fields of vision were counted. All the quantitative data with the confidence intervals (95% CI).

Lysosomal co-localization with nanoprobes

The CT26 cells were seeded into the confocal dishes at a density of 2.5×10^5 cells/dish under 100% humidity, and then cultured at 37 °C in an atmosphere containing 5% CO_2 overnight. Subsequently, the FITC-modified nanoprobes with the Fe^{3+} concentration of

100 mg/L were diluted and added to confocal dishes. After 2 h co-incubation, the supernatant was aspirated and discarded, the Lyso-Tracker Red working solution was added to the cells and incubated for 30 min. Then, the cells were fixed by adding 4% paraformaldehyde. Finally, the nuclear dye DAPI was added and the luminescence images were captured on the laser scanning confocal microscopy (LSCM Leica TCS SP8). Each step calls for thorough rinsing using PBS.

Animal studies

Six-week-old female BALB/c mice were provided by SiPeiFu Biotechnology Co. Ltd. and maintained in SPF level animal room at a temperature of 22±1 °C under a 12-h light-dark cycle and food and water ad libitum.

Animal tumor models

Subcutaneous tumor models were established through inoculating the 4T1 cells (~2.5 $\times 10^6$) into the mice at the flank region of their right hind legs. The tumor MRI studies were carried out at about 7 d after the inoculation of tumor cells.

Biosafety evaluation of nanoprobes

The female BALB/c mice (6-week-old) (n = 3) was randomly chosen intravenously injecting nanoprobes with 10 mg/kg Fe. The other three mice served as the control mice. The body weights were recorded every day for 21 days. At 21 d post-injection, the mice were sacrificed, and the organs such as heart, liver, spleen, lung and kidney were extracted and subjected to H&E staining. In addition, the blood of every mouse was collected and was further performed for blood routine and blood chemical examinations.

Additional Figures

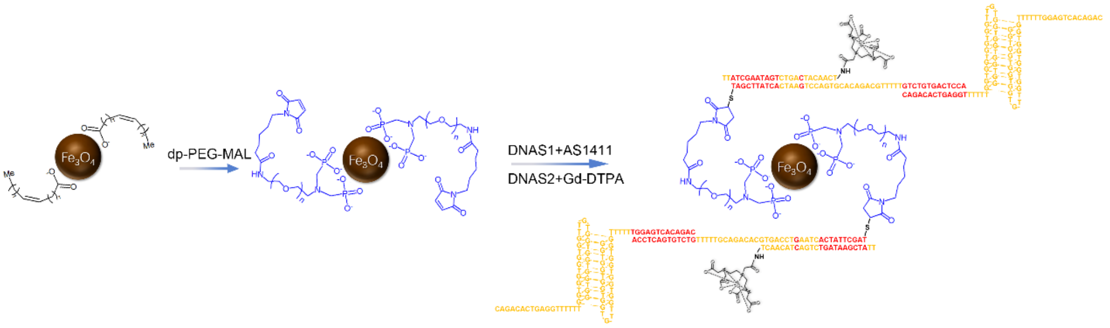

Figure S1. Schematic illustration of the synthetic process of nanoprobes.

Table S1. Sequence of synthesized oligonucleotides.

Name	Sequence (5'-3')
miR-21	UAG CUU AUC AGA CUG AUG UUG A
Control RNA	GUC GUA AAG CCU GAA CUU CAC G
DNAS1	SH-TAG CTT ATC ACT AAG TCC AGT GCA CAG ACG TTT TTG TCT GTG ACT CCA
DNAS2	NH_2-TCA ACA TCA GTC TGA TAA GCT ATT
AS1411	GGT GGT GGT GGT TGT GGT GGT GGT GGT TTT TTG GAG TCA CAG AC
DNAS3	NH_2-CGT GAA GCT AAC TGA TAA GCT ATT
miR-155	UUA AUG CUA AUC GUG AUA GGG GU
miR-10b	UAC CCU GUA GAA CCG AAU UUG U

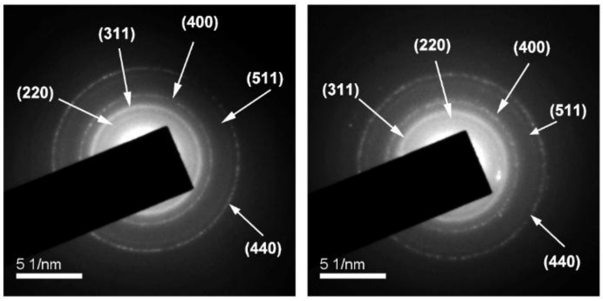

Figure S2. SAED patterns of Fe_3O_4-OA (left) and nanoprobes (right).

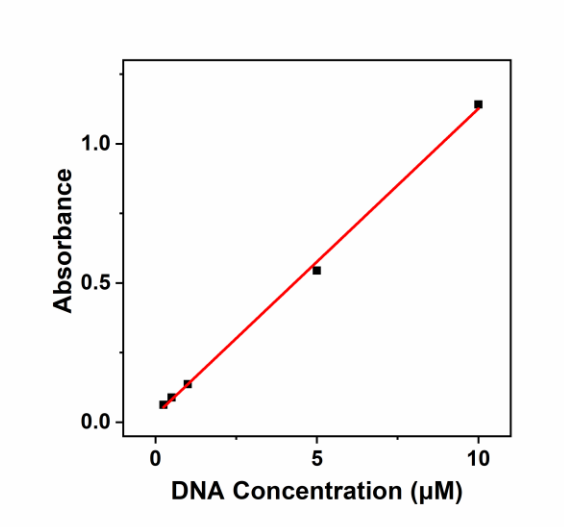

Figure S3. The standard curve of DNAS1+DNAS2+AS1411 at 260 nm.

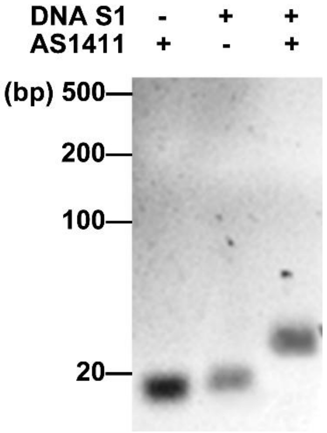

Figure S4. Agarose gel (4%) electrophoresis image of complementary base pairing of DNAS1 and AS1411 aptamer.

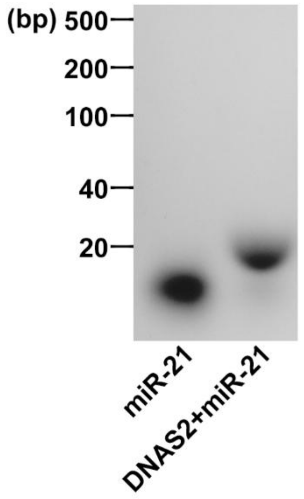

Figure S5. Agarose gel (4%) electrophoresis image of complementary base pairing of DNAS2 and miR-21.

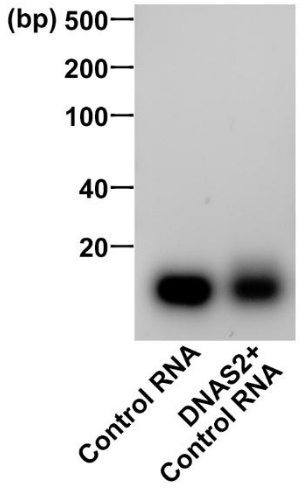

Figure S6. Agarose gel (4%) electrophoresis image of complementary base pairing of DNAS2 and Control RNA.

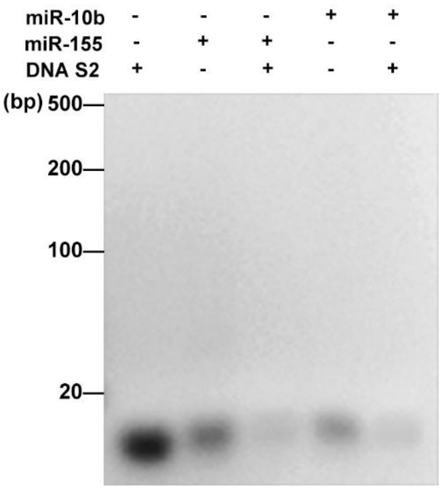

Figure S7. Agarose gel (4%) electrophoresis image of complementary base pairing of DNAS2, miR-155 and miR-10b.

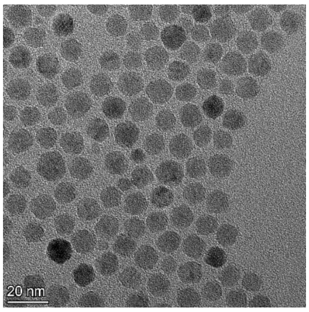

Figure S8. The TEM of nanoprobes after co-incubation with miR-21.

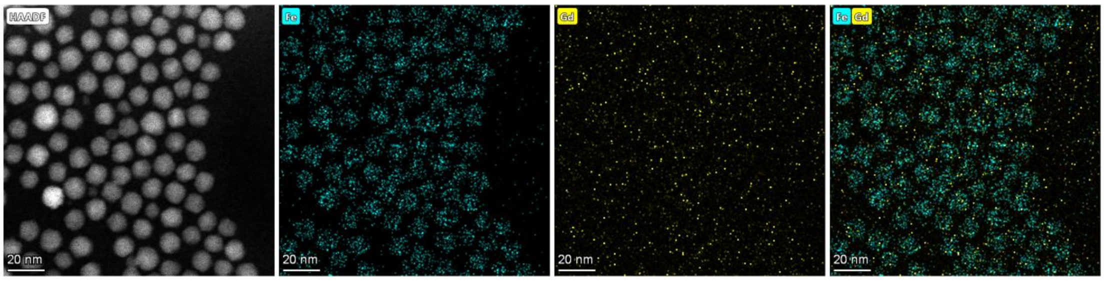

Figure S9. The EDS of nanoprobes after co-incubation with miR-21.

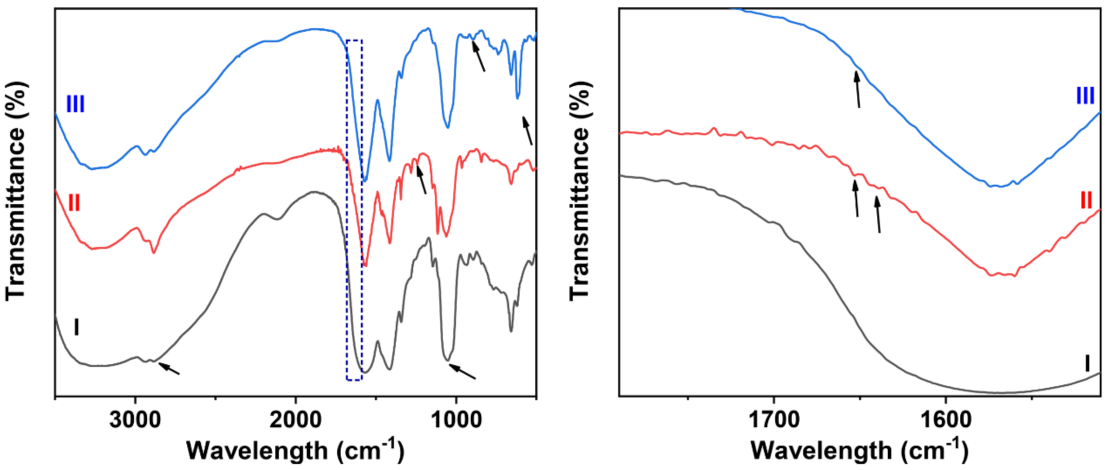

Figure S10. FTIR spectrum of Fe_3O_4-PEG (I), Fe_3O_4-PEG-DNAS1-AS1411 (II) and nanoprobes (III).

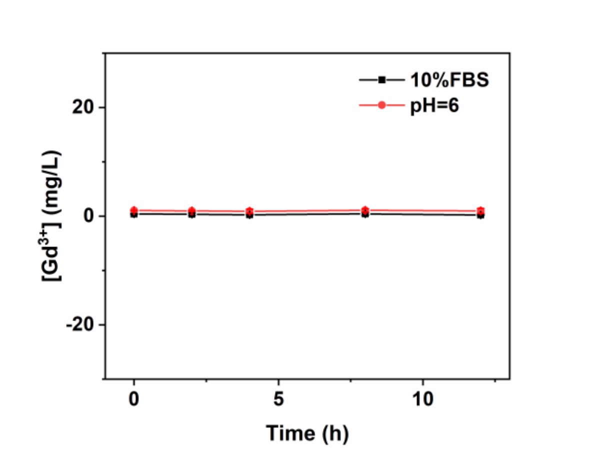

Figure S11. The stability of the nanoprobe in an environment of 10%FBS or pH 6.0.

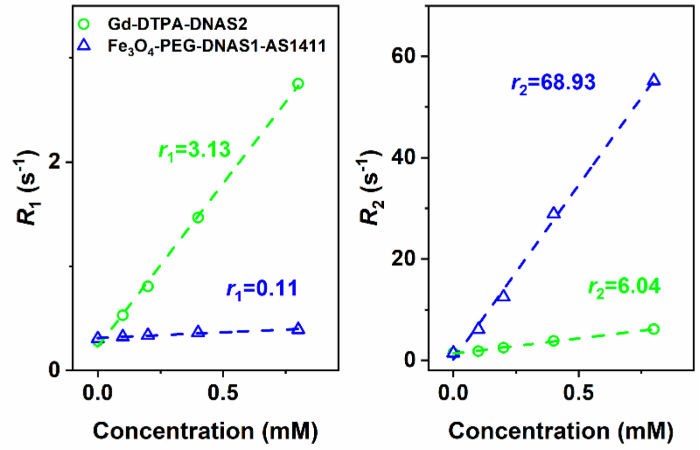

Figure S12. The R_1 and R_2 of Fe_3O_4-PEG-DNAS1-AS1411 and Gd-DTPA-DNAS2, respectively, together with the corresponding linear fittings for extracting r_1 and r_2.

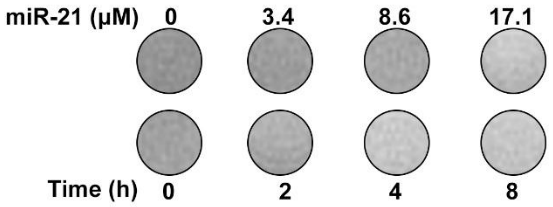

Figure S13. The T_1-weighted images of nanoprobes with different miR-21 concentration and the T_1-weighted images of nanoprobes over time.

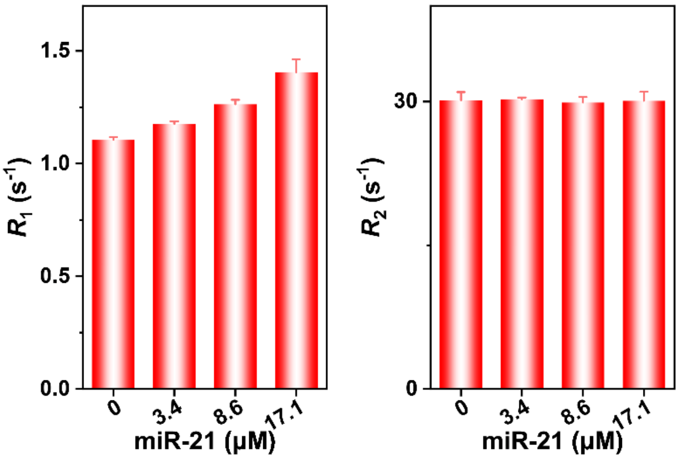

Figure S14. The R_1 and R_2 change of nanoprobes with different miR-21 concentration.

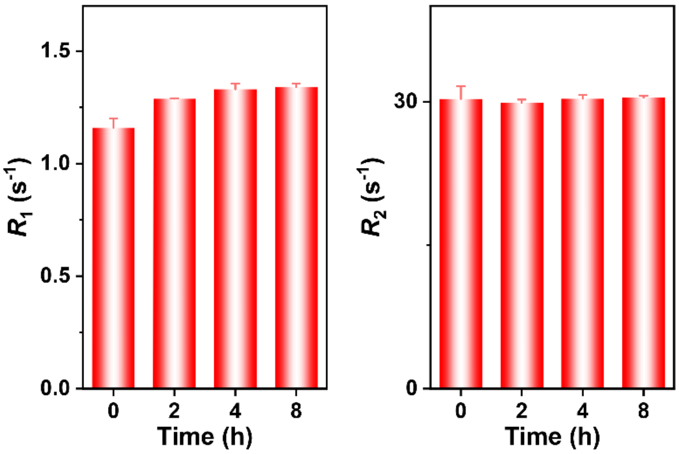

Figure S15. The R_1 and R_2 change of nanoprobes over time.

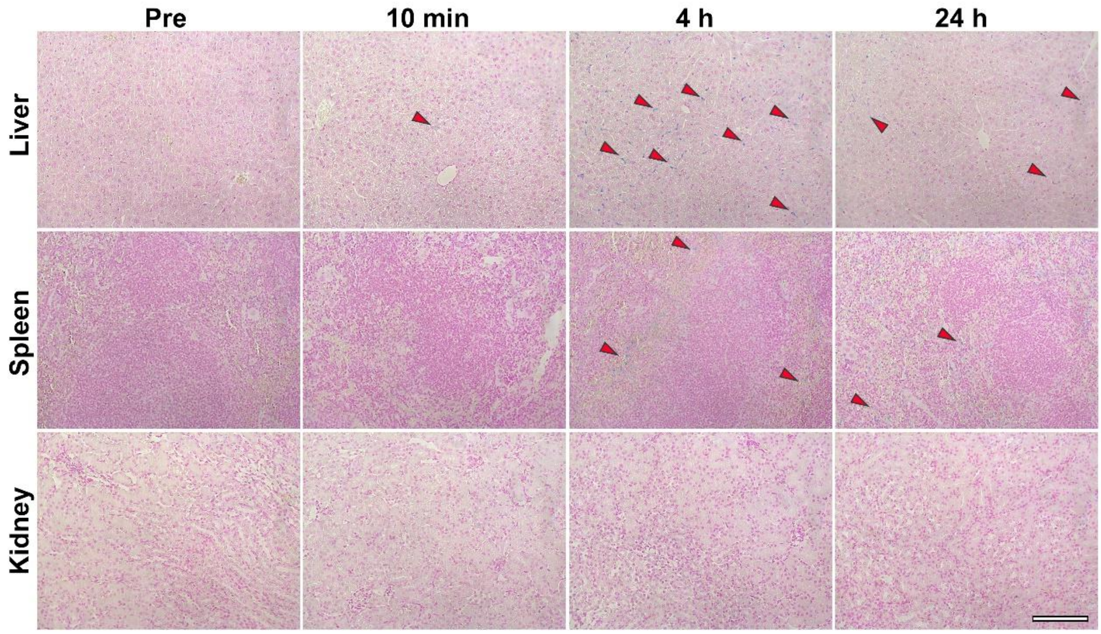

Figure S16. Prussian blue staining at different time point of liver, spleen and kidney of healthy mice (Scale bar: 200 μm).

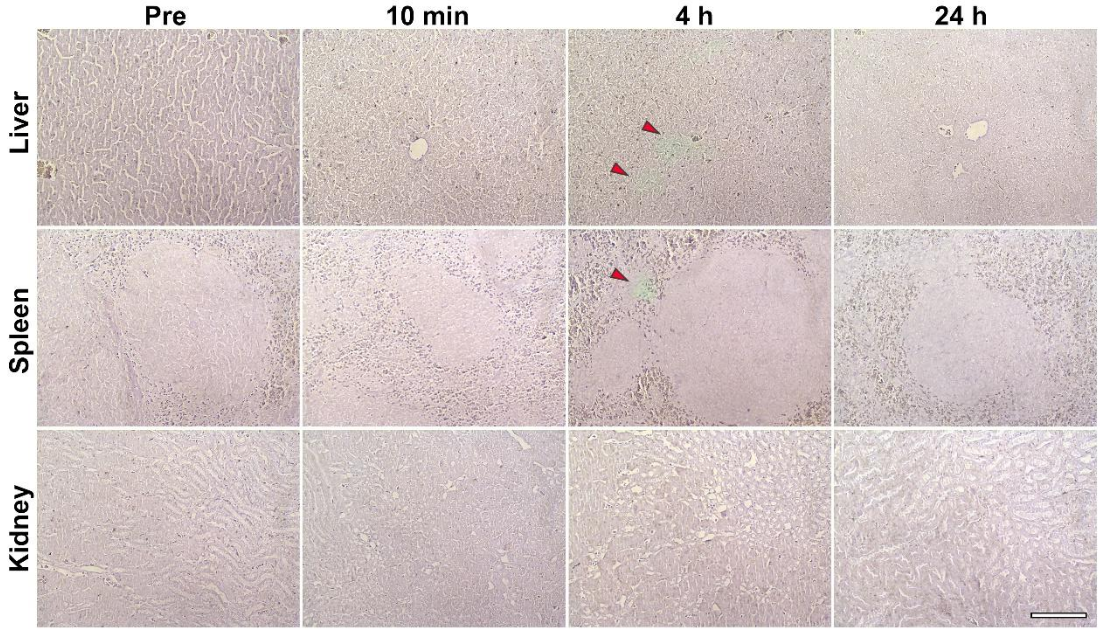

Figure S17. CPN III staining at different time point of liver, spleen and kidney of healthy mice (Scale bar: 200 μm).

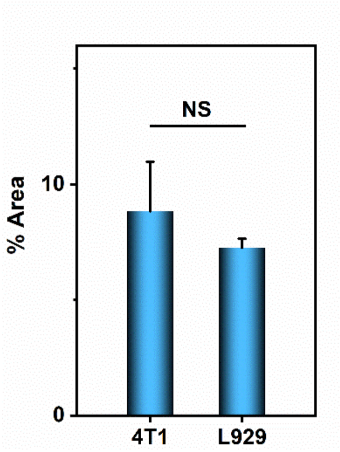

Figure S18. Quantitative analysis of the cell density for LSCM.

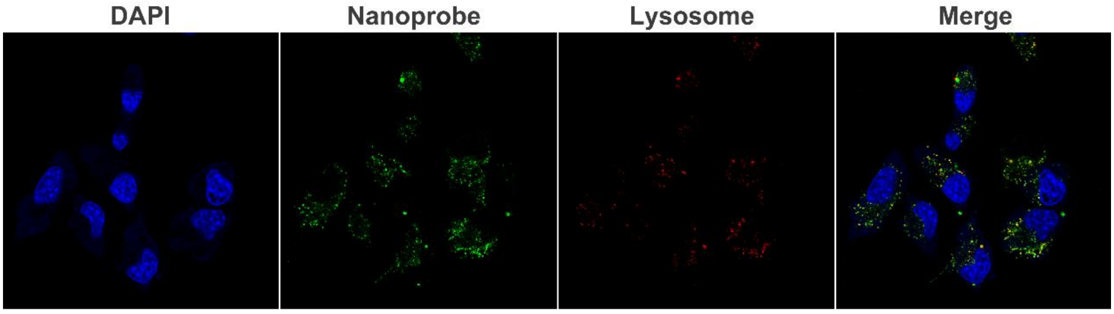

Figure S19. The LSCM analysis of lysosomal (Red) co-localization with nanoprobes (Green).

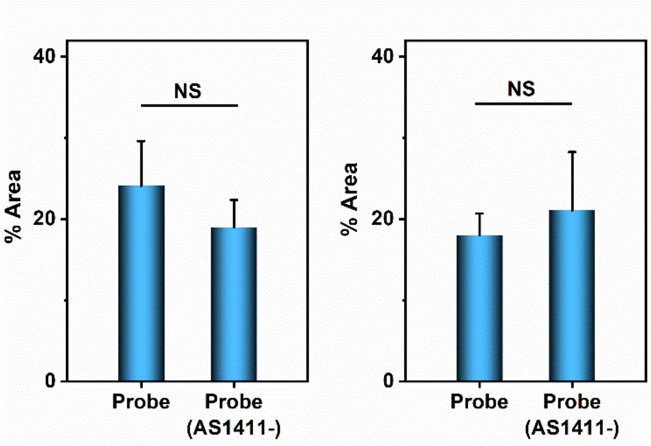

Figure S20. Quantitative analysis of the cell density for Prussian blue staining (Left) and CPN III staining (Right).

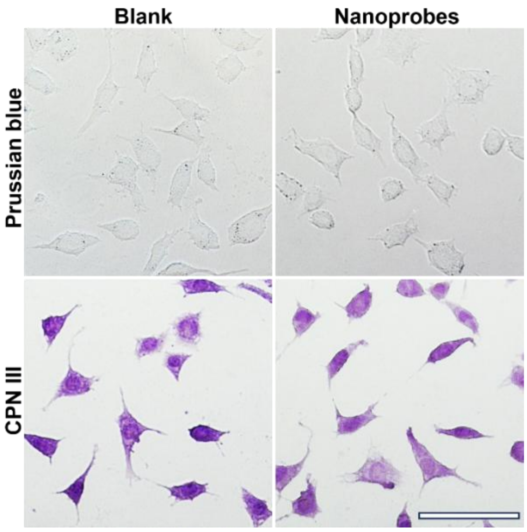

Figure S21. The coincubation of L929 cells with nanoprobes and staining by Prussian blue and CPN III. Scale bar: 100 μm.

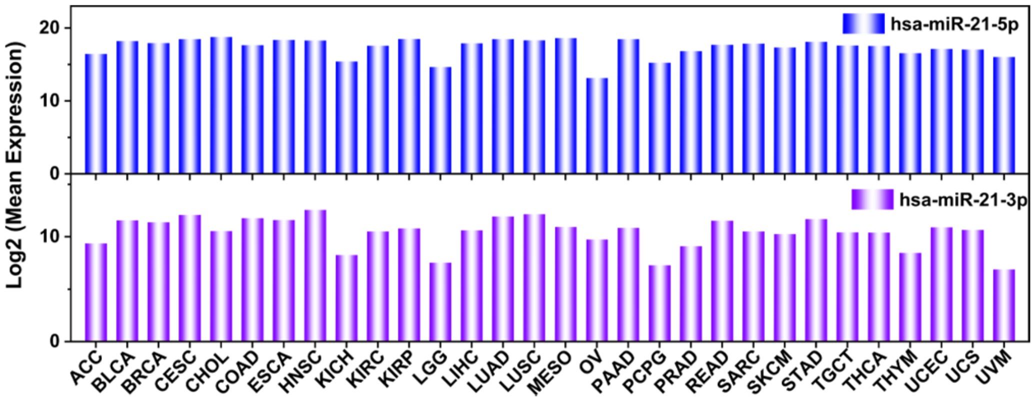

Figure S22. The comparison of miR-21 among different types of tumors. Abbreviation: ACC, adrenocortical carcinoma; BLCA, bladder urothelial carcinoma; BRCA, breast invasive carcinoma; CESC, cervical squamous cell carcinoma and endocervical adenocarcinoma; CHOL, cholangiocarcinoma; COAD, colon adenocarcinoma; ESCA, esophageal carcinoma; HNSC, head and neck squamous cell carcinoma; KICH, kidney chromophobe; KIRC, kidney renal clear cell carcinoma; KIRP, kidney renal papillary cell carcinoma; LGG, brain lower grade glioma; LIHC, liver hepatocellular carcinoma; LUAD, lung adenocarcinoma; LUSC, lung squamous cell carcinoma; MESO, mesothelioma; OV, ovarian serous cystadenocarcinoma; PAAD, pancreatic adenocarcinoma; PCPG, pheochromocytoma and paraganglioma; PRAD, prostate adenocarcinoma; READ, rectal adenocarcinoma; SARC, sarcoma; SKCM, skin cutaneous melanoma; STAD, stomach adenocarcinoma; TGCT, testicular germ cell tumors; THCA, thyroid carcinoma; THYM, thymoma; UCEC, uterine corpus endometrial carcinoma; UCS, uterine carcinosarcoma; UVM, uveal melanoma.

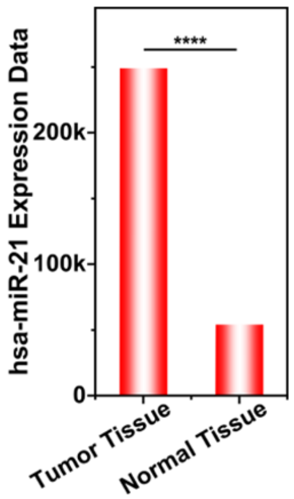

Figure S23. MiR-21 of the normal tissue compared to miR-21 of tumor tissue in breast invasive carcinoma.

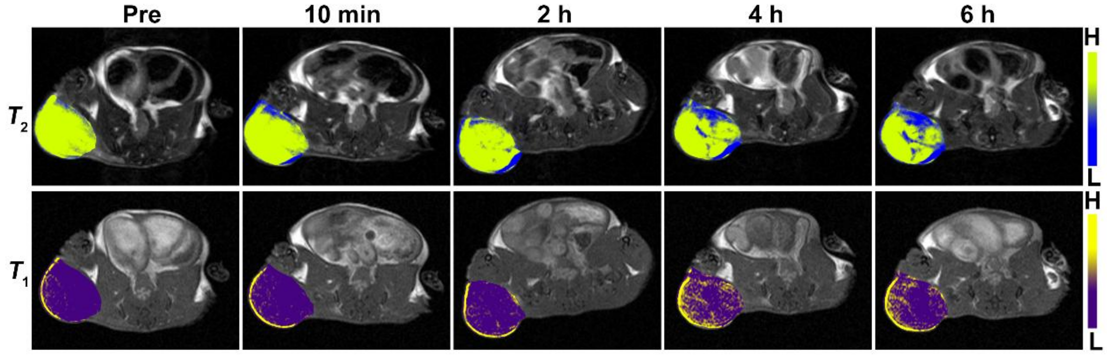

Figure S24. The T_1 - and the T_2 -weighted MR images of CT26 subcutaneous tumor sections acquired before injection and at different time points post-injection of nanoprobes.
